# A quantum physics layer of epigenetics: a hypothesis deduced from charge transfer and chirality-induced spin selectivity of DNA

**DOI:** 10.1186/s13148-023-01560-3

**Published:** 2023-09-08

**Authors:** Reiner Siebert, Ole Ammerpohl, Mirko Rossini, Dennis Herb, Sven Rau, Martin B. Plenio, Fedor Jelezko, Joachim Ankerhold

**Affiliations:** 1 Institute of Human Genetics, Ulm University & Ulm University Medical Center, Albert-Einstein-Allee 11, 89081 Ulm, Germany; 2 Center for Integrated Quantum Science and Technology (IQST) Ulm-Stuttgart, Ulm, Germany; 3 Institute for Complex Quantum Systems, Ulm University, 89069 Ulm, Germany; 4 Institute of Inorganic Chemistry I, Ulm University, 89081 Ulm, Germany; 5 Institute of Theoretical Physics, Ulm University, 89081 Ulm, Germany; 6 Institute for Quantum Optics, Ulm University, 89081 Ulm, Germany

**Keywords:** Epigenetics, Quantum physics, Quantum tunneling, Quantum coherence, Charge transfer, Chirality, Spin, Electron hopping, DNA methylation

## Abstract

**Background:**

Epigenetic mechanisms are informational cellular processes instructing normal and diseased phenotypes. They are associated with DNA but without altering the DNA sequence. Whereas chemical processes like DNA methylation or histone modifications are well-accepted epigenetic mechanisms, we herein propose the existence of an additional quantum physics layer of epigenetics.

**Results:**

We base our hypothesis on theoretical and experimental studies showing quantum phenomena to be active in double-stranded DNA, even under ambient conditions. These phenomena include coherent charge transfer along overlapping pi-orbitals of DNA bases and chirality-induced spin selectivity. Charge transfer via quantum tunneling mediated by overlapping orbitals results in charge delocalization along several neighboring bases, which can even be extended by classical (non-quantum) electron hopping. Such charge transfer is interrupted by flipping base(s) out of the double-strand e.g., by DNA modifying enzymes. Charge delocalization can directly alter DNA recognition by proteins or indirectly by DNA structural changes e.g., kinking. Regarding sequence dependency, charge localization, shown to favor guanines, could influence or even direct epigenetic changes, e.g., modification of cytosines in CpG dinucleotides. Chirality-induced spin selectivity filters electrons for their spin along DNA and, thus, is not only an indicator for quantum coherence but can potentially affect DNA binding properties.

**Conclusions:**

Quantum effects in DNA are prone to triggering and manipulation by external means. By the hypothesis put forward here, we would like to foster research on “Quantum Epigenetics” at the interface of medicine, biology, biochemistry, and physics to investigate the potential epigenetic impact of quantum physical principles on (human) life.

## Introduction

Physiologic and disease development in humans, like in other living creatures, is strongly associated with epigenetic modifications, which link the DNA sequence with responses to external stimuli [1, 2]. Common to all epigenetic mechanisms is that they are informational processes associated with DNA, which, e.g., can regulate gene activity but, by definition, do not alter the DNA sequence itself. The hitherto widely acknowledged epigenetic mechanisms typically rely on chemical processes, including DNA methylation, various modifications of histones, or chromatin remodeling [1].

Nevertheless, besides chemical processes, also physical principles apply to DNA, and quantum physical processes, particularly proton tunneling (for a glossary of quantum physics terms, see Table 1), have long been linked to the generation of DNA mutations. Already Watson and Crick assumed in their landmark paper on the structure of DNA “*that the bases only occur in the structure* [i.e., the double-helix] *in the most plausible tautomeric forms*” [3]. Löwdin put forward a hypothesis in 1963 that tunneling of protons might contribute to the occurrence of mutations in DNA [4]. The concept of proton tunneling in mutation induction has been meanwhile further underpinned by a series of theoretical and experimental modeling studies providing evidence that quantum mechanisms can contribute to mutation induction at ambient conditions [5–8]. Nonetheless, due to energetic reasons, the tautomeric forms induced by quantum tunneling are transient with a short lifespan and, in order not to tunnel back to the original form, need fixation during DNA replication to end up in a bona-fide mutation on a cellular level [5–8]. Thus, though the tautomeric occupation probability has been recently reported as considerably large, the overall quantitative biological impact of proton tunneling in introducing sequence alterations warrants further discussion [8].

**Table 1:** Key physical phenomena discussed in this hypothesis paper

Charge hole:	Is a concept used to describe the movement of positively charged "absence" in a certain material. A hole can be thought of as a missing electron, or a vacancy in the valence band (or HOMO in a molecule) of a material, that behaves as a mobile positive charge carrier. This hole can move through the material as if it were a particle carrying a positive charge
Chirality-induced spin selectivity (CISS):	Phenomenon in which the spin of an electron passing through a chiral molecule (a molecule that is not superimposable on its mirror image) is preferentially oriented in a certain direction. In other words, CISS refers to a dependence of the spin of an electron on the chirality of a molecule through which it passes. This effect has been observed in a variety of organic molecules, including amino acids, DNA, and proteins
Coherence/decoherence:	Quantum coherence is a property of quantum systems that refers to the ability of different quantum states to interfere with each other, resulting in a pattern of constructive and destructive interferences. In contrast, decoherence refers to the loss of coherence in a quantum system due to its interaction with its environment. As a quantum system interacts with its surroundings, the coherence of the system can be disrupted, causing the quantum system to behave more like a classical system
Coupled harmonic oscillators:	A harmonic oscillator is a system that exhibits simple harmonic motion, such as the motion of a mass on a spring. When two or more harmonic oscillators are coupled, meaning they interact with each other in some way, the resulting system is known as a harmonic chain. The interaction between oscillators can lead to the formation of collective excitations (known as phonons), energy transfer between oscillators, and, for quantum oscillators, the creation of entangled states
Exciton:	In physics, an exciton is a bound state of an electron and a positively charged "electron hole" that are attracted to each other by the Coulomb force. When an atom or molecule is illuminated by an external source, such as light, an electron can be excited to an energetically higher lying state. This creates an electron–hole pair, where the vacancy (hole) is the lower energy state. This pair of charges then interacts to form a so-called exciton
Proton tunneling:	Also known as “proton transfer,” is a quantum mechanical phenomenon where a proton (H +) moves through an energy barrier that it would not be able to overcome according to classical physics. In the case of proton tunneling, a proton can move through a barrier, such as a hydrogen bond, to form a new (covalent) bond with another atom or molecule. In DNA replication, proton tunneling has been proposed as a mechanism for ensuring that the correct nucleotide bases are paired together, helping to prevent mutations
Quantum delocalization/localization:	Quantum delocalization refers to the spread-out nature of a quantum particle's wave function over a larger region of space. This phenomenon can also be related to wave-like superposition. Quantum localization, on the other hand, refers to the confinement or localization of a quantum particle within a small region of space
Superexchange:	Transfer of electrons via quantum tunneling from a donor to an acceptor through an intermediate and energetically higher lying ‘bridge’. The concept of superexchange can be applied to biological systems, particularly in the context of electron transfer reactions in proteins and enzymes
Tautomer:	Is a type of isomer, a molecule with the same chemical formula as another molecule, with a different arrangement of atoms, specifically isomers that differ in the placement of a proton (H +) and the double bond within the molecule

## Hypothesis

In contrast to the impact of quantum effects on mutational mechanisms, the role of quantum physical principles in epigenetics has not yet been deeply interrogated [9, 10]. Nevertheless, as discussed in detail below, theoretical modeling and experimental studies provide compelling evidence for a range of physical phenomena leading to altered properties of DNA molecules beyond mutation and chemical modification. External factors can trigger these physical phenomena and potentially influence gene expression. Thus, we here hypothesize (I) that a layer of epigenetics driven by quantum physical processes in DNA exists; (II) that two (potentially interacting) quantum physical processes (not exclusively) contributing to this layer of epigenetics are (a) coherent charge/exciton transfer along DNA and (b) chirality-induced spin selectivity by DNA (see Table 1); (III) that the quantum physical layer of epigenetics can interfere with and is not erased by well-recognized chemical epigenetic layers like DNA and histone modification or chromatin structure; and (IV) that the quantum physical layer acts under ambient cellular conditions.

## General considerations for the hypothesis

**Fig. 1:**
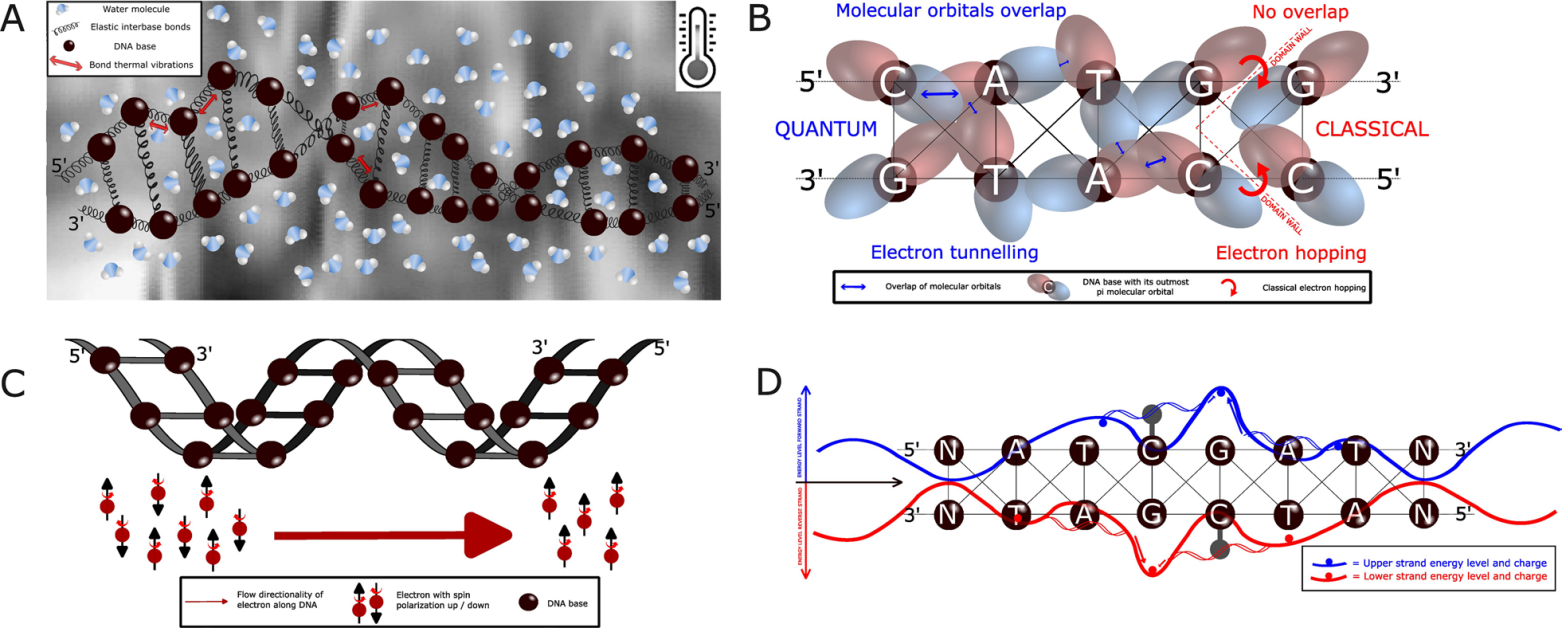
**A** Schematic visualization DNA as harmonic oscillator in a cell. Far from being a rigid, isolated stairwise structure of subsequent nucleobases, DNA is considered as an elastic complex, in constant interaction with surrounding molecules (indicated by water molecules in the example) and the thermal excitations (indicated by grey temperature gradient) coming from its background. **B** DNA with stacking pi-orbitals allowing electron tunneling. Schematic representation of DNA double strand. Each sphere represents a nucleotide along the strands, surrounded by its Lowest Unoccupied Molecular Orbital (LUMO) populated by an excited electron. The overlap of close by orbitals (blue arrows) allows the electron to move from a base to the other through quantum tunneling phenomena. Eventual lack of orbital overlap (domain walls) prevents this phenomenon to happen, resulting in the electron only managing to populate the neighboring base through classical hopping processes, thermally enhanced. In this representation, we use pi-orbitals as example of LUMO as in the literature there are many references to these orbitals as being the most suitable for our modeling. **C** Chirality-induced spin selectivity by DNA. The chiral structure of the DNA helix is such that electrons with opposite spin are pushed in different directions along the chain, resulting in an effective quantum spin selectivity phenomenon within the DNA structure. **D** Guanine as charge trap in DNA. The dynamics of an excited electron along the chain (decided by the energy landscape characterizing the strand) is usually dragged toward sites containing guanine bases. The cytosine on the opposite strand of the guanine is prone to epigenetic modification (e.g., methylation) in a CpG context. Whereas cytosine methylation itself does not discontinue charge transfer (but might change its dynamics), flipping out the base for modification in the enzymatic process can perturb charge transfer

We are building our hypotheses on the fact that DNA is a chiral molecule with a vibrating and, in general, dynamical structure (rather than a rigid structure as might be suggested by the classical Watson–Crick model) (Fig. 1A) [3, 11–16]. Moreover, it is known that in a double-helical structure of a B-DNA with proper base pairing, the pi-orbitals of neighboring bases of a strand tend to overlap. Thus, electron clouds (i.e., delocalized negative charges) shared by neighboring DNA bases can be formed (Fig. 1B) [17–23]. This tendency is counterbalanced by the vibrating dynamics of DNA and its backbone which typically suppresses delocalization. Between these clouds, charge transfer and charge separation can be induced by a tunneling mechanism or classical hopping, which leaves a (positively) charged hole on the donor base [17–22]. The quantum tunneling, including superexchange phenomena, between spatially separated areas differs from thermally-induced (i.e., non-quantum) hopping in that the latter relies on overcoming classical energy barriers for electron (charge) movement (Fig. 1B) [17–22, 24–26]. Finally, it must be recognized that at physiological temperatures, DNA is embedded in an environmental "bath" consisting of ionic and dipolar molecules (e.g., water) that can interact with DNA at a quantum level by altering the geometrical and electrical properties of the DNA structure [27, 28], thereby also suppressing quantum coherence (Fig. 1A, Table 1) [29–33].

## Supporting evidence for the hypothesis

### Charge and exciton transfer in DNA

On a theoretical level, we and others investigated the quantum diffusion of single charges, excitons, and the relation to spin selectivity along and across both strands of DNA under ambient (physiologic) conditions using tight-binding lattice models representing the two-strands of DNA embedded in thermal environments mimicking solvent and/or residual molecular degrees of freedom (backbone) (Fig. 1C) [34, 35 Rossini et al., in preparation]. Such models predict that even at moderate temperatures coherence (tunneling) of charges over a few nucleobases may play an important role depending on the energetic profile determined by the sequences and on proper conformations of neighboring bases. Strong evidence exists that the intra-strand quantum features may survive on relatively long-time scales compared to bases-intrinsic electronic processes. In addition, a series of investigations using a broad range of experimental designs has documented relatively large charge mobility over rather long molecular distances (up to 34 nm, i.e., over up to 100 bases, corresponding to a picoseconds time scale for charge dynamics) in DNA [17–22]. An efficient charge transfer prerequisite is proper stacking of the bases and double-stranded DNA ensuring proper stacking of pi-orbitals [17–22, 36, 37]. Perturbation of the DNA stack by e.g., mismatches, some DNA binding proteins, or conformational changes hampers charge transfer, thus, creating “sections” in the DNA defined through their differential charge transfer potential [38–40]. In line with the transfer along the pi-orbitals of the nucleobases in the long axis of the DNA, perturbation of its sugar DNA backbone seems not to affect charge transfer, arguing for a sequence-related charge transfer pattern in DNA [37]. Current data suggest the coexistence of two mechanisms of charge transfer along DNA: coherent tunneling over short distances of 3 to 4 bases fostered by vibrating movements of nucleobases with overlapping pi-orbitals and incoherent (thermal) classical hopping between domains of such well-coupled stacked bases [17–22]. Charge transfer over even longer distances (4 nm) is facilitated by the interaction of these base stacks triggered by external light. Further, light-induced electron transfer events between redox active metal complexes bound to DNA suggest strong participation of the extended pi-orbital network of DNA [40]. In sum, these charge dynamics lead to shorter (coherent) and longer distance (incoherent) charge transfer, which change the electrostatic properties of regions in the DNA [17–22, 41]. Besides altering the structure of the DNA (e.g., kinks, bends), such local charge imbalances can potentially affect proper sequence and/or conformation-dependent binding of proteins and, thus, the regulation of transcriptional processes [38, 41, 42]. This may be particularly true in a cell environment under oxidative stress [21]. In reverse, protein binding has been shown to affect charge transfer, which can be exploited for diagnostic purposes [43]. Moreover, recent reports indicate that human DNA primase utilizes electron transfer through dsDNA to determine the DNA replication activity [44]. Notably, there seem to exist different probabilities for certain genomic domains to be involved in charge transfer processes, thus more likely displaying local charge imbalances [11, 17–22, 45]. Whether such physically “cold” and “hot” DNA areas transfer into special biological properties warrants further investigation.

### Chirality-induced spin selectivity by DNA

Chirality is a geometric property of certain biological molecules, including DNA, describing that such molecules and their mirror images are non-superimposable. A physical property of chiral molecules is that the transfer of electrons also depends on their spin degree of freedom, e.g., by filtering electrons with different spin orientations when passing through those molecules such that an accumulation of electrons with the same spin orientation appears in certain areas (Fig. 1C) [46]. The in-depth physical principles underlying this so-called chirality-induced spin selectivity have yet to be wholly understood [47–49]. Remarkably, spin dependent charge transfer leading to spin polarization and charge transfer through double-stranded DNA have been experimentally documented indicating that charge transfer and chirality-induced spin selectivity are interconnected physical principles in DNA. The impact of this connection on chemical or structural DNA properties, however, requires further investigation. Findings describing that spin-dependent charge transfer is enhanced through oxidatively damaged DNA, which, as discussed above, is less conductive in charge transfer, might indicate that the spin-selectivity could also be linked to the otherwise more insulating sugar backbone or even the water spine of DNA [50]. CISS might also reduce the coherent backscattering of electrons resulting in enhanced charge transfer through DNA. Whatever the mechanisms are, spin selectivity can e.g., affect spin-sensitive interactions of DNA [51, 52].

### Interaction of the quantum physical with chemical epigenetics layers

At least for charge transfer in DNA, it has been experimentally shown that it is not prevented or grossly hindered by DNA methylation or binding to histones [39, 50]. Moreover, if protein binding to DNA does not affect proper base stacking, it preserves coherent charge transfer [38]. Thus, classical chemical mechanisms do not principally prevent quantum physical epigenetic effects. On the other hand, some chemical epigenetic processes might interfere with physical phenomena: binding of proteins, like TBP, transcription factors, CTCF, or cohesins, which kink, bend, or otherwise affect the pi-orbital stacking disrupt charge transfer [38, 53, 54]. Remarkably, prominent examples of proteins disrupting proper pi-stacking are many writers, readers, and erasers of the modified DNA bases (e.g., a series of DNA methylases and demethylases), which flip a target nucleobase within the DNA for their recognition and/or modification [55]. Hence, charge transfer is attenuated by such epigenetic modifiers [38, 56]. In turn, it has not escaped our attention that the guanine base has been identified in our and also in other models as a charge trap (holes) as guanine is the most easily oxidized DNA base (Fig. 1D) [17, 20–22, 57, Rossini et al. in preparation]. Consequently, charge holes (i.e., a positive charge) are more stable on a G:C base pair, an effect even enhanced in guanine doublets and triplets named “charge sinks”. Considering that the cytosine paired with the guanine in both strands of the CpG dinucleotide is the preferred base of DNA modification, it is intriguing to speculate that charge transfer, charge delocalization, and charge separation are involved in directing or facilitating this epigenetic change. Besides altering the DNA structure or spin-selective binding of proteins, many other effects of quantum physical changes in the DNA can be envisioned to change the transcriptional activity of the genome. Nevertheless, these yet lack experimental evidence.

### Action of a quantum physical layer under ambient cellular conditions

For decades, it has been postulated that time scales for decoherence are too short for quantum mechanics to apply to macromolecules under ambient conditions, like DNA. Nevertheless, as outlined above, current models taking into account physiological temperatures and charge decoherence by the environment as well as experimental findings at corresponding conditions and using DNA in solution (and even embedded in histones), suggest quantum effects to be active in DNA in living cells. Vice versa, DNA in living cells is exposed to a wealth of environmental intracellular and extracellular physical stimuli, which can trigger and modify the processes outlined above, ranging from the radiation of various wavelengths and magnetic fields up to reactions providing electrically charged radicals. A quantum physics layer of epigenetics may help to explain the physiologic responses to such threads.

## Conclusion and outlook

In summary, we think there is sufficient evidence for postulating a quantum physics layer of epigenetics, which we propose to name “Quantum Epigenetics". Our hypothesis on this layer's existence, along with some features, is built on theoretical and experimental data from many groups. Remarkably, whereas physics principles in DNA have been extensively explored until the early years of this millennium, research on quantum effects in cellular DNA seems thereafter to have mainly focused on mutational mechanisms. It seems intuitive to us that the more volatile epigenetic landscape is much more susceptible to the transient and partly stochastic quantum effects. These, in turn, might be susceptible to induction or influence by external stimuli, as proposed for irradiation [58]. The principles outlined herein offer, on one hand, the opportunity to further understand how information in DNA is bringing the phenotype (including diseases) into being and how this can be influenced by external factors. As an additional part, charge transfer in DNA–RNA hybrids and quantum features of RNA molecules might extend the present hypothesis toward more epitranscriptomic aspects [59, 60]. On the other hand, the charge transfer and spin properties can be explored for (epi)genetic and functional diagnostics, as it has already been documented for charge transfer and DNMT1 activity [43]. Finally, the physical principles outlined above are, in principle, prone to external manipulation, e.g., in the context of treatment.

## Data Availability

Not applicable.

## Abbreviations

CISS: Chirality-induced spin selectivity
CTCF: CCCTC-binding factor
DNA: Desoxyribonucleic acid
HOMO: Highest occupied molecular orbital
LUMO: Lowest unoccupied molecular orbital
TBP: TATA box binding protein
